# Hypogonadotropic hypogonadism presenting with arhinia: a case report

**DOI:** 10.1186/1752-1947-7-52

**Published:** 2013-02-22

**Authors:** Jeanie B Tryggestad, Shibo Li, Steven D Chernausek

**Affiliations:** 1 Department of Pediatrics, Section of Diabetes and Endocrinology, 1200 Children’s Way, Suite 4500, Oklahoma City, OK 73104, USA; 2 Department of Pediatrics, Section of Genetics, 1200 Children’s Way, University of Oklahoma Health Sciences Center, Oklahoma City, OK 73104, USA

## Abstract

**Introduction:**

Arhinia, congenital absence of the nose, is a rare malformation. We present the third reported case of arhinia accompanied by hypogonadism and demonstrate that this is due to gonadotropin deficiency.

**Case presentation:**

A 13-year-old Caucasian boy with congenital arhinia presented for evaluation of delayed puberty and micropenis. We examined genes known to be associated with hypogonadotropic hypogonadism for mutations and performed a chromosomal microarray to assess copy number variation.

**Conclusion:**

No mutations in *KAL1*, *FGFR1*, *PROK2*, *PROKR2*, *FGF8*, *CHD7* and *GnRHR* were identified in our patient and there were no copy number variations observed that would explain the phenotype. Though studies are limited in such patients, we suggest that hypogonadotropic hypogonadism is associated with arhinia and that the two entities likely result from a common genetic cause that affects early nasal development and gonadotropin-releasing hormone neuron formation or migration.

## Introduction

Arhinia, congenital absence of the nose, is a rare congenital malformation. Only 36 cases have been reported in the literature to date, but most reports have focused on the surgical reconstruction of the nose with little attention to other hormonal defects associated with the malformation [1]. Although a few cases have been attributed to chromosomal abnormalities, most are idiopathic [2-6]. Features associated with arhinia include microphthalmia, choanal atresia and cleft palate [1-5]. A single report describes hypogonadism in two male patients with arhinia [7], with only a single genetic locus evaluated as a mechanistic link between the two conditions. Mutations in genes known to be involved in nasal development have also been sought, but none of the reported cases of arhinia have been associated with such mutations [1,3,7].

The association of anosmia (absence of the sense of smell) and hypogonadotropic hypogonadism is known as Kallmann syndrome and is far more common than arhinia [8-12]. Hypoplasia of the olfactory bulbs is a frequent finding and other midline defects occur as well. Mutations in *KAL1*, *FGFR1* and other genes have been shown to lead to these conditions, but the majority of cases have no identifiable cause [8-12].

The development of the nose and the origin and migration of the cells destined to become the hypothalamic neurons controlling gonadotropin secretion are embryonic-related events. Thus, in the case presented, we sought a genetic cause by examining in detail the structure of genes known to be associated with hypogonadotropic hypogonadism and/or Kallmann syndrome as well as by a genome-wide assessment of copy number variation.

## Case presentation

A 13-year-old Caucasian boy with congenital arhinia presented for evaluation of delayed puberty and micropenis. He was born at 32 weeks gestation weighing 2045g. The absence of his nose resulted in significant respiratory distress at birth necessitating resuscitation and subsequent tracheostomy, which was maintained until age 2 years. He also had a cleft lip and palate that were repaired in infancy. He had no other dysmorphic features, notably no microphthalmia. He met developmental milestones appropriately. No family history of arhinia, anosmia, delayed puberty or hypogonadism was reported.

Absence of the nose along with mid-face hypoplasia was the most striking feature on physical examination. His height was 161.6cm (50^th^ percentile for age and sex). His weight was 79.9kg (greater than the 97^th^ percentile for age and sex). He appeared of normal intelligence and was taking advanced placement classes in school. On genital examination, his left testicle could not be palpated. His right testis was 2mL in volume and descended into his scrotum. The stretched penile length was 3.7cm (two standard deviations below the mean for his age). His pubic hair was Tanner stage 2. Subsequent to this examination, orchiopexy of his left testicle had been attempted, but on exploration the testis was noted to be small and of poor quality, resulting in orchiectomy.

Our patient’s bone age was 14 years by the standards of Greulich and Pyle [13] at chronological age 13 years and 10 months, which is slightly advanced for hypogonadotropic hypogonadism, but could be explained by his obesity. Computed tomography imaging with three-dimensional reconstructions of his facial bones revealed a lack of nasal bones, the absence of sinus cavities and lacrimal glands, and maxillary hypoplasia.

A Smell Identification Test was administered; this consists of four booklets containing 10 scratch and sniff odorants. The test is derived from basic psychological test measurement theory and focuses on the comparative abilities of individuals to identify a number of odorants at the supra-threshold level [14]. The individual’s score of correctly identified odorants is compared to scores of normal individuals of the same age and gender using tables provided in the administration manual. Only 11 of 40 possible smells were identified, diagnostic of anosmia as the test is designed such that someone unable to smell would guess the correct answers 25% of the time.

Serum was obtained for measurements of gonadotropin levels and testosterone and whole blood for gene testing. His karyotype was 46,XY with no structural or numerical anomalies. Gonadotropin and testosterone measures at 13 years of age were consistent with severe hypogonadotropic hypogonadism. His level of luteinizing hormone was <0.1IU/L (expected range for age, 0.2 to 5.0IU/L) and follicle-stimulating hormone was 0.3IU/L (expected range for age, 1.2 to 5.8IU/L). At 13 years of age, his testosterone measured 1.1nmol/L (expected range for pre-pubertal males <0.7 to 0.8nmol/L). A pathological examination of the extirpated gonad revealed tissue consistent with atrophic testis.

His DNA was screened for rare sequence variants in the genes *KAL1*, *FGFR1*, *PROK2*, *PROKR2*, *FGF8* and *GnRHR* by Dr William Crowley at the University of Massachusetts. These genes were selected for examination because of their known association with Kallmann syndrome and/or hypogonadotropic hypogonadism. Rare sequence variants were defined as DNA sequence variations with minor allele frequency of less than 1% in normal healthy controls. Screening for rare sequence variants in the *KAL1*, *FGFR1*, *PROK2*, *PROKR2*, *FGF8* and *GnRHR* genes were all negative. Sequencing for the coding region of *CHD7* was provided by Dr. Shibo Li at the University of Oklahoma Health Sciences Center. No mutations were identified.

Array comparative genomic hybridization was carried out with a commercially available array designed to detect copy number imbalances (losses or gains) across the whole genome (NimbleGen 720K V.3.1, Roche NimbleGen System Inc., Madison, WI, USA). The array contains 720,000 oligonucleotides with a median probe space of approximately 2.5kb that represents coding and noncoding human sequences in the genome (content sourced from the University of California, Santa Cruz hg18 human genome: March 2006 National Center for Biotechnology Information build 36, RefSeq and GenBank databases). One normal female validated DNA sample was used as an opposite sex control for the analysis. Data interpretation was based on information from University of California, Santa Cruz Genome Browser [15], the Database of Genomic Variants [16] and an internal copy database. A high sensitivity microarray was carried out with specific attention to the loci of the known genes involved in hypogonadotropic hypogonadism and nasal development including *MSX1*, *NRP2*, *ALX3* and *ALX4.* No chromosomal imbalances of these regions were seen on this analysis.

At age 13 years and 11 months, treatment with intramuscular testosterone enanthate at 75mg a month was begun and increased to the full adult replacement dose over 29 months. With testosterone replacement, his penile length increased to 9cm, pubic hair advanced to Tanner stage 4, acne developed and he experienced an overall improved sense of well-being. At the age of 17, he is in a staged process of building a nose.

## Discussion

In this case report, we describe what we believe to be the third case identified of arhinia with hypogonadism. In contrast to the previous report [7], we have documented hypogonadotropic hypogonadism with paired testosterone and gonadotropin levels. Though it is assumed that the two patients previously reported by Graham and Lee [7] also had hypogonadotropic hypogonadism, gonadotropin levels were not measured; therefore, primary hypogonadism was not excluded. McGlone reported the case of a child with micropenis and low basal gonadotropin levels measured at an undefined age, but the levels were normal upon luteinizing hormone-releasing hormone stimulation [4]. One other very early report by Lutolf described a male patient with arhinia and bilateral undescended testicles, but no other biochemical evaluation [17]. Also in the first case reported by Graham and Lee [7], no genetic testing was performed, and in the second case, only a karyotype with fluorescent *in situ* hybridization for mutations in *KAL1* was obtained. In our case, we have performed an extensive investigation into the genes associated with hypogonadotropic hypogonadism as well as copy number. Lastly, both previously reported cases by Graham and Lee were classified as Bosma syndrome, an association of arhinia with microphthalmia [7]. Our patient does not have Bosma syndrome, and therefore, the genetic basis may differ from our case.

Embryonic development of the nose is complex. Beginning at the fourth week of gestation, the frontonasal processes appear and slowly elevate into the dorsum and apex of the nose [5]. During the fifth week of gestation, the nasal placodes invaginate forming the nasal pits [5]. The pits deepen and mesenchyme in the margins of the pits gives rise to the medial and lateral nasal processes [5]. The medial processes fuse to form the nasal septum and medial aspect of the upper lip while the lateral processes form the alae of the nose [5].

In embryonic development, neurons that produce gonadotropin-releasing hormone (GnRH) migrate along the olfactory neurons, which act as axonal guides, from their origin at the nasal placode to the hypothalamus [10]. The vomeronasal nerve travels from the nasal epithelium, crosses the cribriform plate, and then sends fibers dorsally to the olfactory bulb [9]. Fibers of the caudal vomeronasal nerve travel ventrally to the hypothalamus along with the GnRH-producing neurons [10]. Several factors have been identified that aid in GnRH neuronal migration, including anosmin-1, fibroblast growth factor signaling, neural crest adhesion molecules, and nasal embryonic luteinizing hormone-releasing hormone factor; however, the exact regulation of the migratory process is unknown [9].

Based on the current understanding of nasal and GnRH neuron development, we would anticipate that patients with arhinia would also have congenital hypogonadotropic hypogonadism. Given that hypogonadotropic hypogonadism is much more common than arhinia and several genetic mutations have been documented in cases of hypogonadotropic hypogonadism, we began by looking for mutations in the known genes. Because no mutations were identified in the genes known to cause hypogonadotropic hypogonadism, we proceeded to a genome-wide array to look for copy number variants; however, this revealed no abnormalities that might be attributed to arhinia and hypogonadotropic hypogonadism.

We hypothesize that the phenotype of our patent results from inadequate development of the nasal placode or related structures that interfere with either development or migration of GnRH neuron precursors. The genes directing these events presumably act at a much earlier point in development than those related to Kallmann syndrome and its variants. Although we found no gene abnormalities, point mutations or small deletions that significantly alter gene expression could still be present, but would be missed by chromosomal microarray.

## Conclusion

Arhinia is a rare congenital malformation that to date has not been associated with an identifiable genetic abnormality. While we did not find a genetic explanation with our current analysis, it is still very likely that the two entities are a result of a common genetic cause. It is possible in the future that, as new genetic loci for hypogonadotropic hypogonadism and nasal development are identified, the exact mechanism will become apparent.

## Consent

Written informed consent was obtained from the patient and consent was obtained from the legal guardian of the patient for publication of this case report. A copy of the written consent is available for review by the Editor-in-Chief of this journal.

## Abbreviations

GnRH: gonadotropin-releasing hormone.

## Competing interests

The authors declare that they have no competing interests.

## Authors’ contributions

JBT conducted the background research for the presentation as well as collecting all the pertinent clinical details for the presentation. JBT also drafted the manuscript. SL conducted the microarray analysis and *CHD 7* mutation assay. SDC helped with interpretation of the data and preparation of the manuscript. All authors read and approved the final manuscript.
